# Supplementary material for: pykarambola: Minkowski tensor morphometry of 3D structures
Source: Bioinform Adv. 2026 Sep 17;6(1):vbag273. doi: 10.1093/bioadv/vbag273 (PMC13668341; doi:10.1093/bioadv/vbag273)
Supplement: Supplementary file 1 [file vbag273_supplementary_data.pdf]

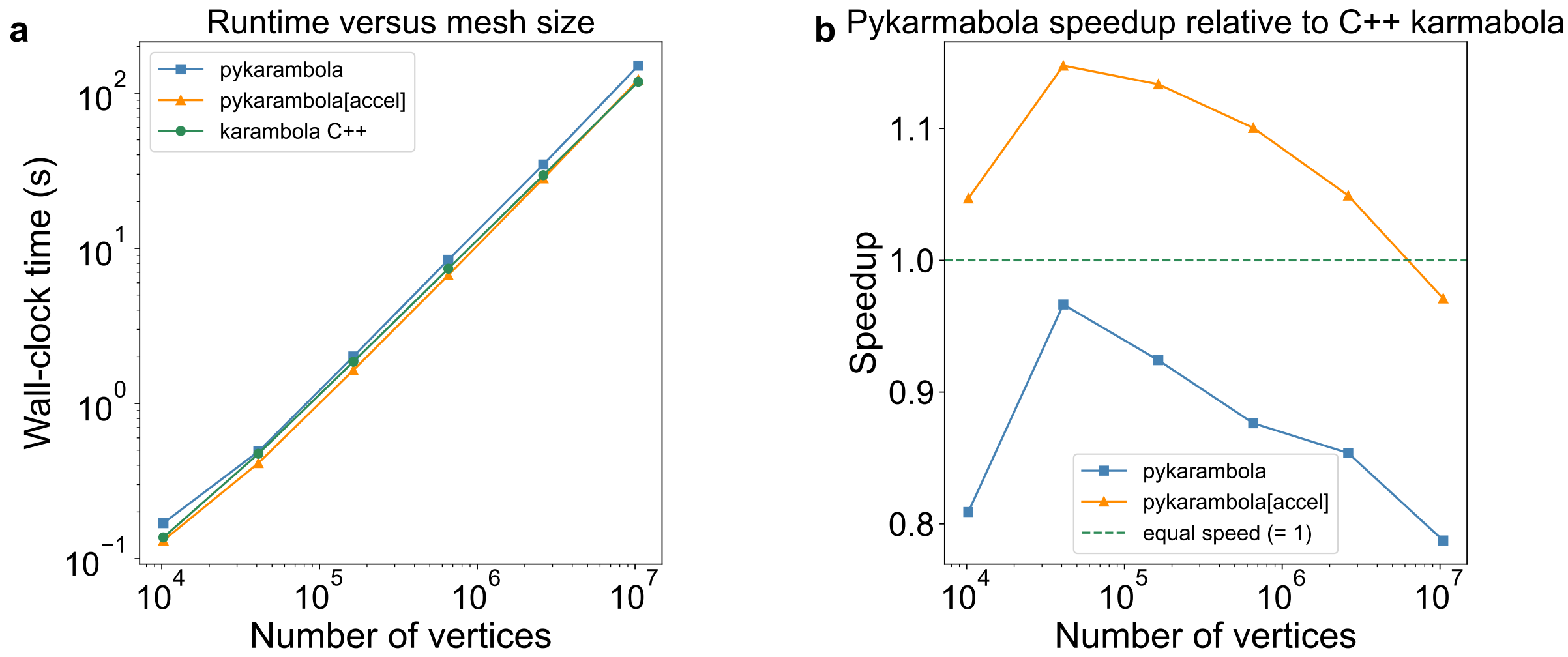

Figure S1: Runtime benchmark on synthetic icospheres (subdivision levels 5–10; 10,242–10,485,762 vertices). **Left**: absolute wall-clock time (log-log scale) for end-to-end execution (mesh parse + Minkowski tensor compute) for C++ karambola (green), pykarambola pure-Python (blue), and pykarambola Cython-accelerated (orange). **Right**: speedup ratio (karambola time / pykarambola time); values above the dashed line (= 1) indicate pykarambola is faster. *Alt text: two-panel figure. Left panel: log-log line plot of wall-clock time versus mesh vertex count for three implementations (karambola, pure-Python, Cython); all three scale similarly with the Cython build closest to or faster than karambola. Right panel: speedup ratio versus vertex count; pure-Python line sits below 1 at small sizes and approaches 1 at large sizes; Cython line stays at or above 1 across the full range.*

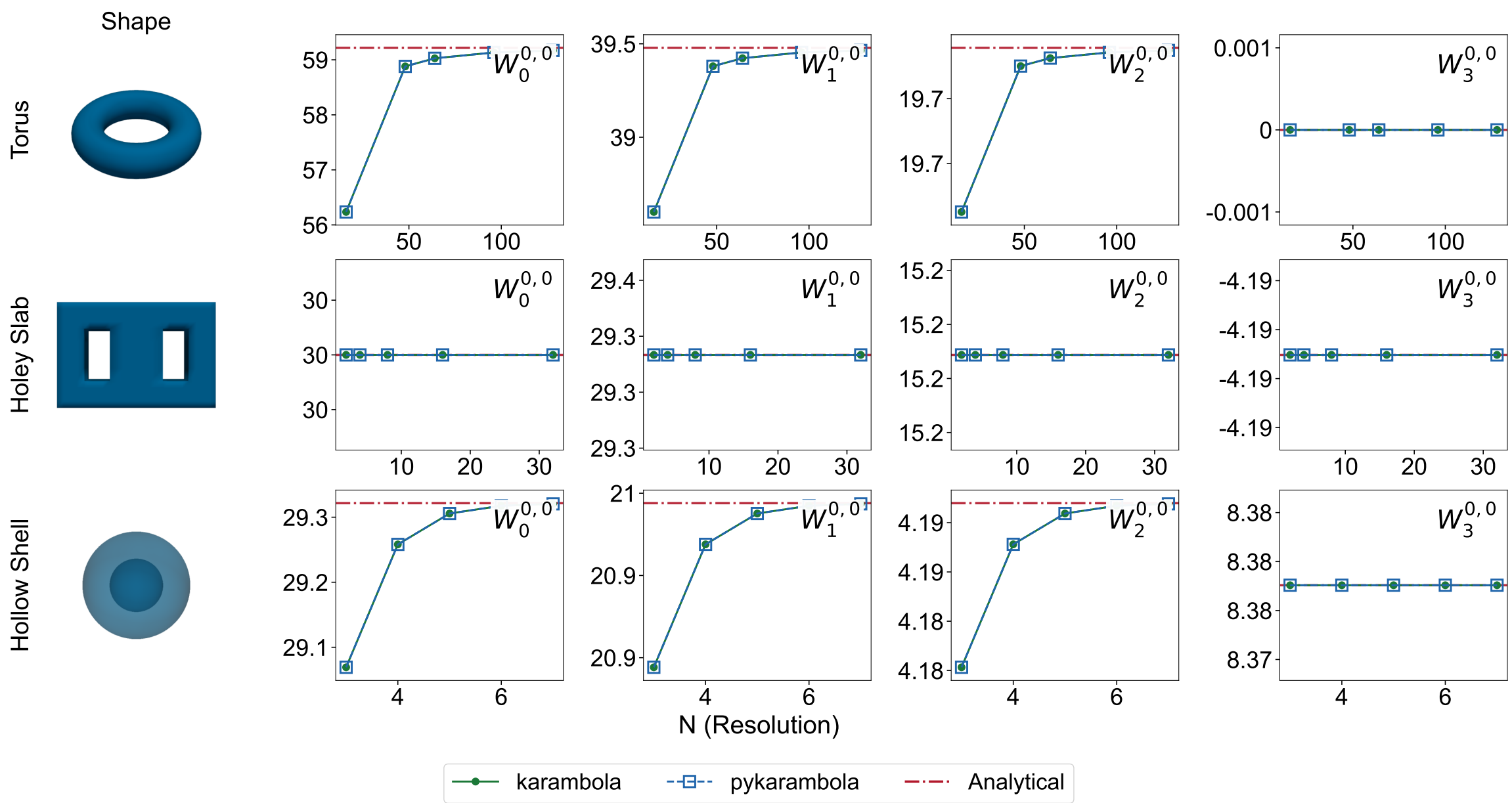

Figure S2: Resolution convergence of `pykarambola` and C++ `karambola` toward analytical Minkowski scalar values on three synthetic meshes of known topology. Each plot panel shows one scalar Minkowski functional against mesh resolution n for karambola (green, solid, filled circles), pykarambola (blue, dashed, open squares), and the analytical value (red dash-dot line). The leftmost column (*Shape*) shows a representative 3D rendering of each mesh. Rows correspond to shapes: torus ($\chi = 0$, genus 1), holey slab (rectangular slab with two rectangular through-holes, $\chi = -2$, genus 2), and hollow shell (two nested icospheres, $\chi = +4$, two components). The four plot columns correspond to the four scalar Minkowski functionals: volume $W_0^{0,0}$, surface area $W_1^{0,0}$, integrated mean curvature $W_2^{0,0}$, and the topological term $W_3^{0,0}$ ($\propto \chi$). Both implementations converge toward the analytical values with increasing resolution and agree with each other to near floating-point precision at every resolution; $W_3^{0,0}$ is a topological invariant and is reproduced exactly at all resolutions. Each shape was evaluated at five mesh resolutions. *Alt text: a grid of panels with three rows (torus, holey slab, hollow shell). The leftmost column shows a 3D rendering of each shape; the remaining four columns are line plots of the volume, surface area, integrated mean curvature, and topological scalar Minkowski functionals against mesh resolution. In each line plot a green solid curve (karambola) and a blue dashed curve (pykarambola) nearly coincide and approach a red dash-dot horizontal line marking the analytical value as resolution increases, with residual differences at the level of floating-point precision. The topological-term panels are essentially flat on the analytical value at all resolutions.*

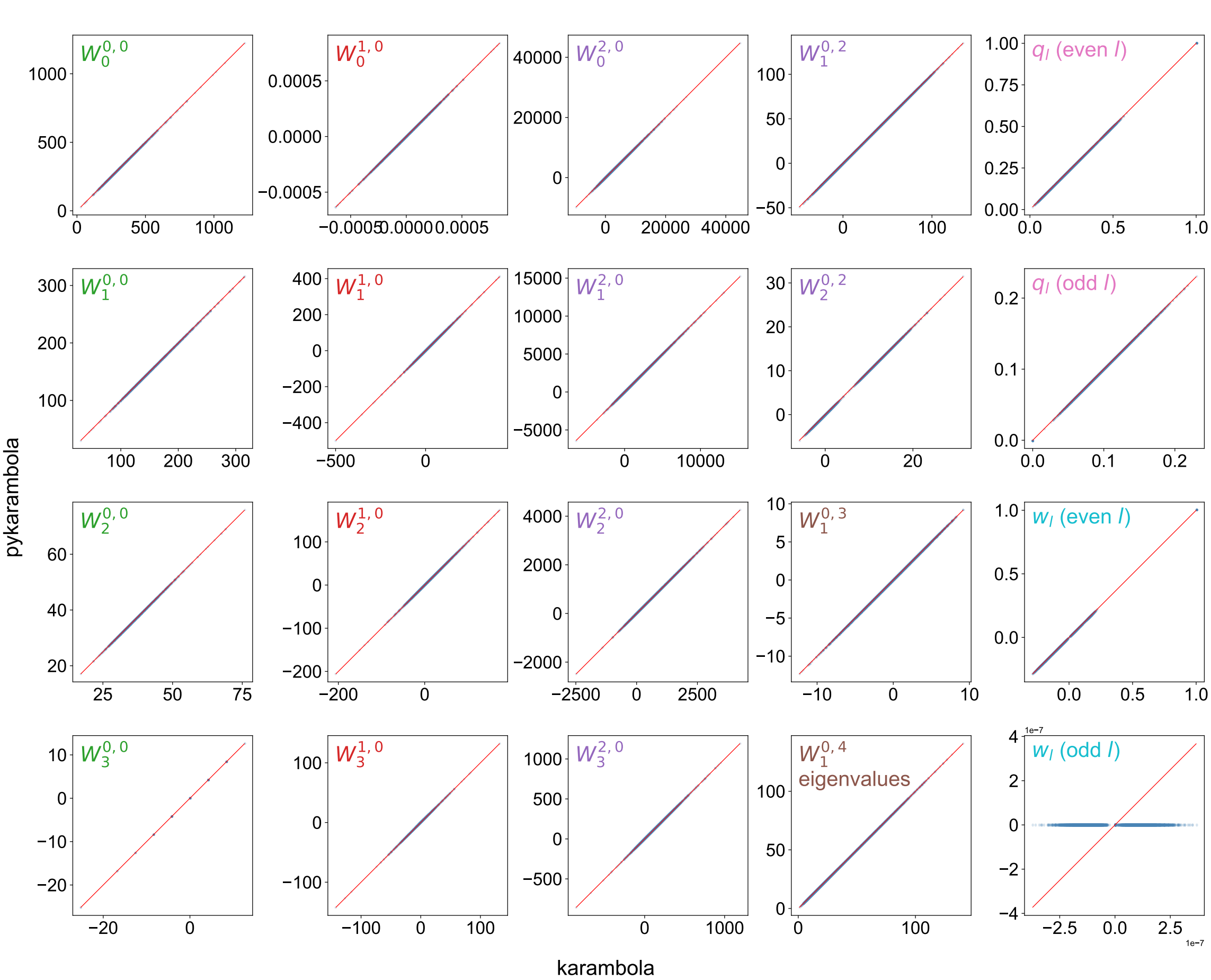

Figure S3: Numerical accuracy of pykarambola relative to C++ karambola. Each panel shows a scatter plot of values from identical analyses run in C++ (karambola, x-axis) and Python (pykarambola, y-axis) on the AdrenalMNIST3D dataset (1,584 meshes, 28^3 voxel resolution). Columns correspond to feature groups: scalars ($W_\nu^{0,0}$), vectors ($W_\nu^{1,0}$), rank-2 tensors ($W_\nu^{2,0}$, $W_\nu^{0,2}$), and higher-rank quantities ($W_1^{0,3}$, $W_1^{0,4}$ eigenvalues, and spherical Minkowski summary quantities q_l and w_l). Panel labels are shown in the top-left corner of each panel, colored by feature group. The red diagonal indicates perfect agreement; all points fall on this line except odd-l `msm_wl`, which pykarambola returns as exactly zero while karambola produces small spurious residuals (see text). Equivalent agreement is observed at 64^3 resolution (Supplementary Figure S4). *Alt text: a grid of scatter plots with four columns (scalars, vectors, rank-2 tensors, higher-rank and MSM quantities) and multiple rows, one per feature. The x-axis shows C++ karambola values and the y-axis shows pykarambola values; a red diagonal marks perfect agreement. All scalar and tensor panels show points tightly on the diagonal; odd-l msm_wl panels show pykarambola at exactly zero while karambola has small non-zero residuals.*

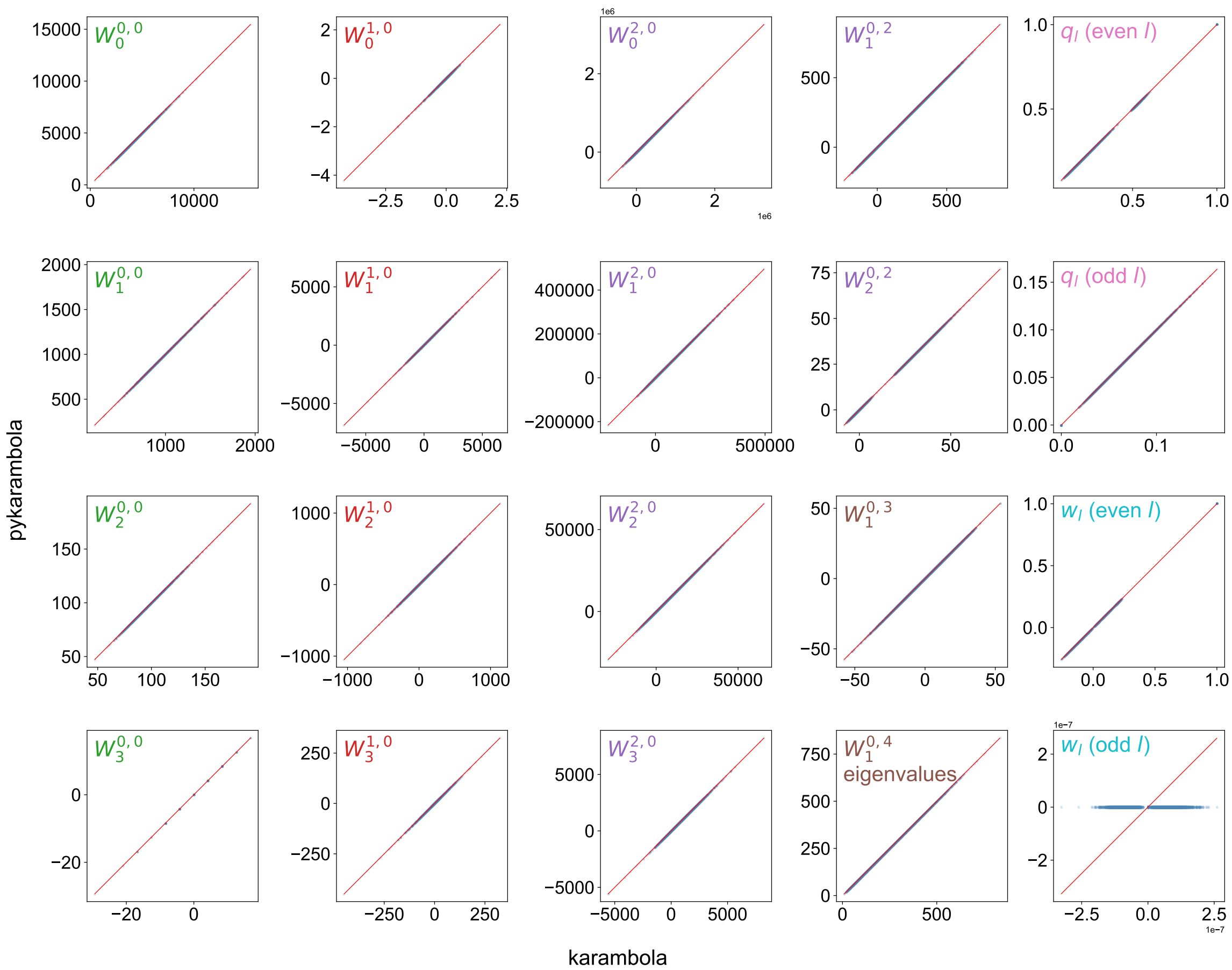

Figure S4: Numerical accuracy of pykarambola relative to C++ karambola at 64^3 voxel resolution. All panels and groupings are identical to Supplementary Figure S3. *Alt text: same layout as Supplementary Figure S3 at* 64^3 *voxel resolution; all points lie on the red diagonal indicating perfect agreement between the two implementations.*

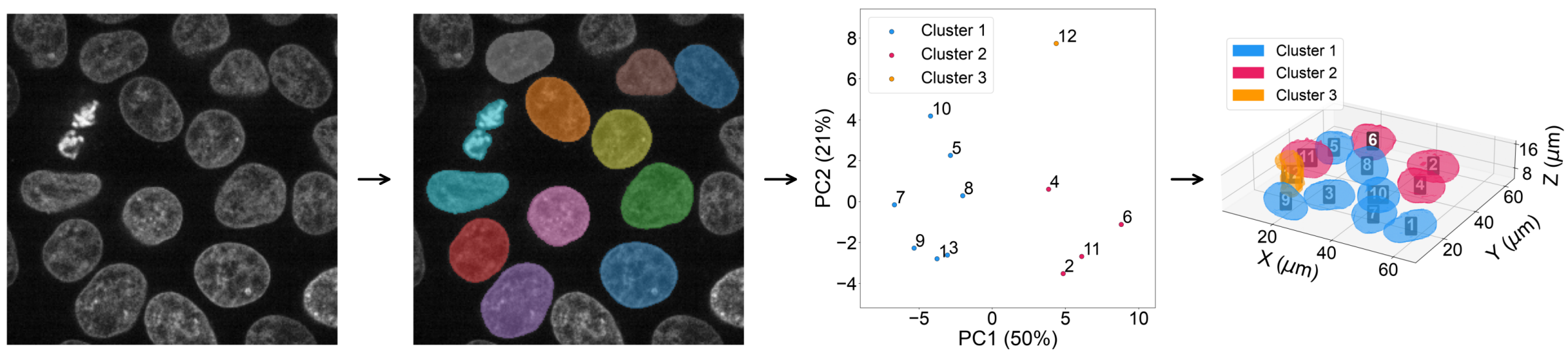

Figure S5: End-to-end segmentation-to-morphometry pipeline applied to 3D confocal nuclei (`skimage.data.cells3d` [van der Walt et al., 2014]). From left to right: (i) raw fluorescence stack (nuclei channel), mid-Z slice shown; (ii) integer label image produced by scikit-image watershed segmentation, each colour representing one of 12 individually resolved non-border-touching nuclei; (iii) PCA projection of the rotationally invariant subset of tensor outputs (Minkowski scalars, β, eigenvalues, trace, and MSM q_l/w_l; 56 features per nucleus) computed in a single `minkowski_tensors_from_label_image` call, points coloured by hierarchical agglomerative cluster assignment; (iv) 3D surface meshes of all nuclei coloured by the same cluster assignment; the spatially isolated nucleus (orange) is the mitotic cell (lower β, larger volume). The complete pipeline is provided in `examples/segmentation_workflow.ipynb`. *Alt text: four-panel horizontal figure connected by arrows showing a left-to-right pipeline. First panel: grey-scale confocal mid-Z slice. Second panel: colour-coded integer segmentation overlay. Third panel: 2D PCA scatter plot with three coloured clusters. Fourth panel: 3D mesh render with nuclei coloured by cluster; one orange nucleus is spatially isolated.*

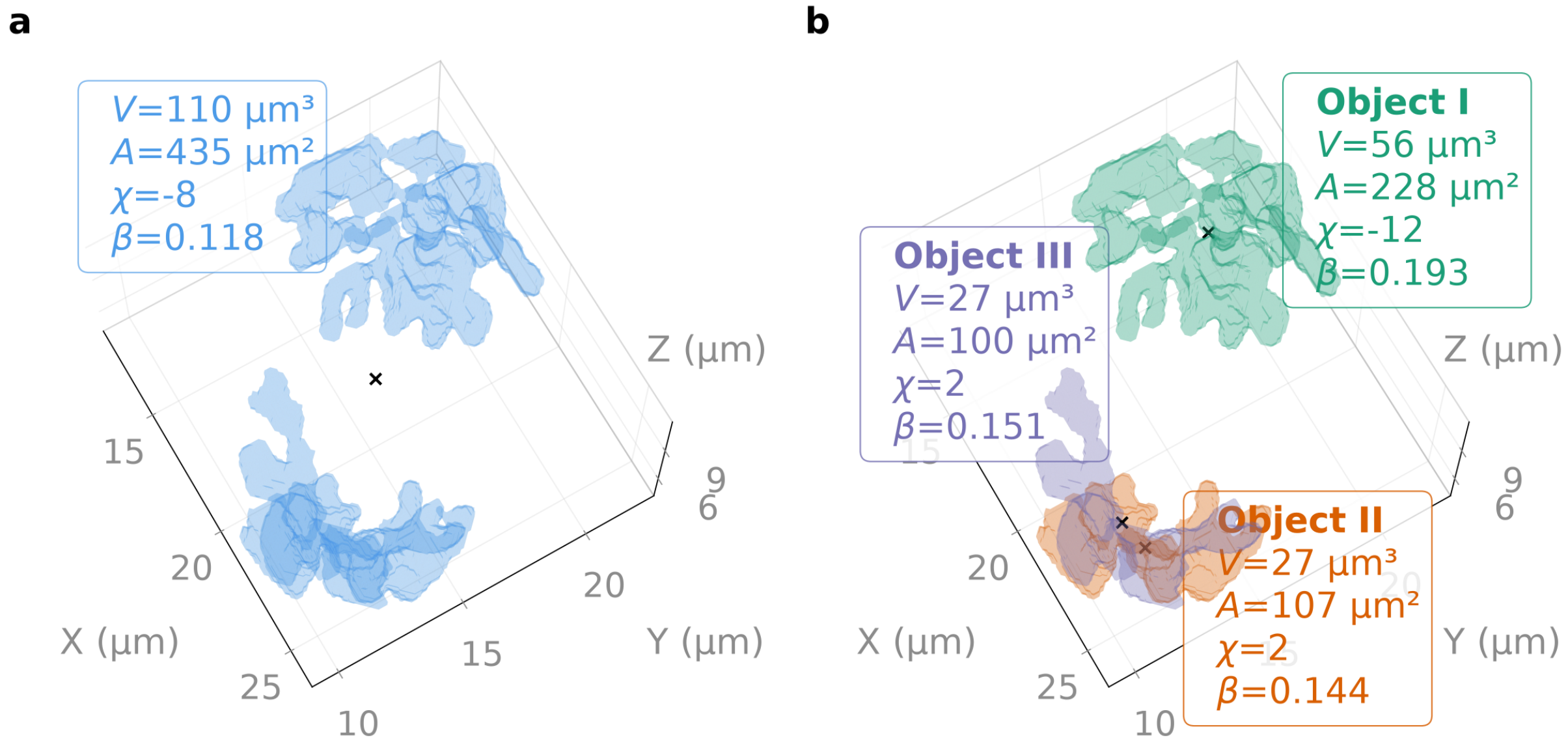

Figure S6: Label-image API applied to the DNA channel of a 3D fluorescence microscopy segmentation of a hiPSC in anaphase/telophase (Allen Cell Collection [Viana et al., 2023]; isotropic voxel size 0.108 μm). Per-object Minkowski tensors report volume (V), surface area (A), Euler characteristic (χ), and anisotropy index (β), with object centroids marked ($\times$). (**a**) The entire DNA channel assigned as a single label; one tensor set describes the aggregate of all three DNA objects ($V = 110\,\mu\text{m}^3$, $A = 435\,\mu\text{m}^2$, $\chi = -8$, $\beta = 0.118$). (**b**) The binary DNA mask passed with `autolabel=True`; pykarambola detects three connected components internally and returns three independent tensor sets, one per DNA object (Object I: $\chi = -12$; Objects II–III: $\chi = 2$ each). Objects 2 and 3 occupy non-overlapping axial ranges (Z slices 40–72 and 78–110, respectively) and are likely segments of the same daughter chromosomal mass. *Alt text: two 3D mesh renders of chromosomal DNA. Left panel (a): a single blue semi-transparent mesh of the aggregate chromatin mass, annotated with V, A, chi, and beta values. Right panel (b): three separately coloured meshes for individual DNA objects, each annotated with its own V, A, chi, and beta values and a centroid cross marker.*

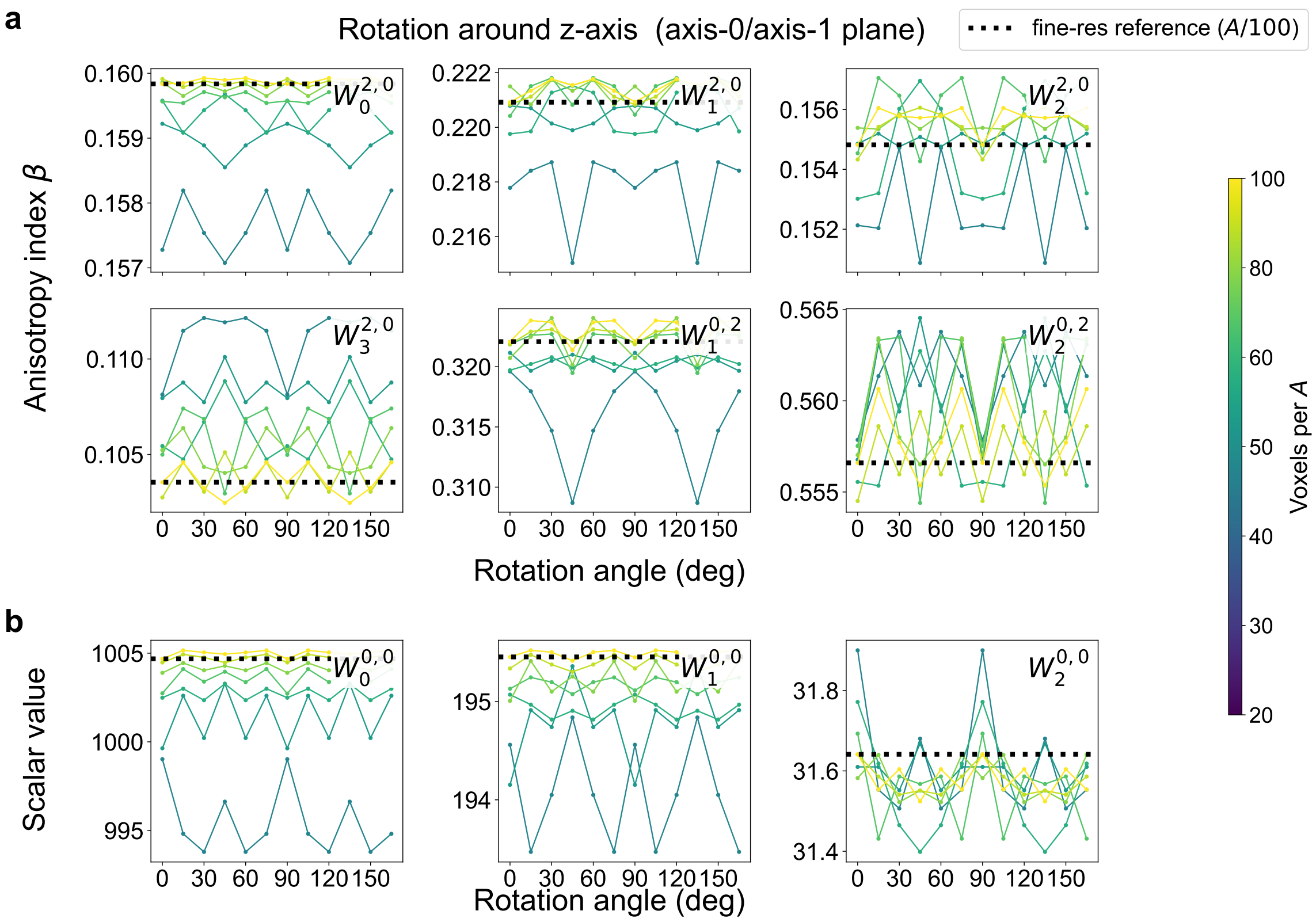

Figure S7: Orientation dependence of Minkowski measures under grid rotation. A synthetic triaxial ellipsoid (semi-axes $A = 10$, $B = 6$, $C = 4$, in arbitrary distance units) was rotated about the z-axis through 12 orientations (0°–165° in 15° steps) at seven grid resolutions (20–100 voxels per semi-axis A; colour scale). Each curve traces one measure against rotation angle at fixed resolution; the periodic variation with angle is the orientation-induced discretisation artefact, and its amplitude decreases with increasing resolution as the curves converge toward the dotted reference. The dotted black line is the fine-resolution reference, evaluated at the finest grid (100 voxels per A) at 0° orientation. (**a**) Anisotropy index $\beta = \lambda_{\min}/\lambda_{\max}$ (ratio of smallest to largest tensor eigenvalue) for the six rank-2 Minkowski tensors $W_0^{2,0}$, $W_1^{2,0}$, $W_2^{2,0}$, $W_3^{2,0}$, $W_1^{0,2}$, $W_2^{0,2}$. (**b**) Value of the three scalar Minkowski functionals $W_0^{0,0}$ (volume), $W_1^{0,0}$ (surface area), and $W_2^{0,0}$ (integrated mean curvature); the topological functional $W_3^{0,0}$ is omitted as it is an integer invariant unaffected by orientation and resolution. *Alt text: a nine-panel figure, six panels in (a) and three in (b), each plotting a morphometric measure against rotation angle from 0 to 165 degrees, with one coloured line per grid resolution from 20 (dark) to 100 (light) voxels per semi-axis. All curves oscillate periodically with angle; the oscillation amplitude shrinks and the curves approach a dotted black reference line as resolution increases.*

References

Stéfan van der Walt, Johannes L Schönberger, Juan Nunez-Iglesias, François Boulogne, Joshua D Warner, Neil Yager, Emmanuelle Gouillart, Tony Yu, and the scikit-image contributors. scikit-image: image processing in Python. *PeerJ*, 2:e453, 2014. doi:10.7717/peerj.453.

Matheus P Viana, Jianxu Chen, Theo A Knijnenburg, Ritvik Vasan, Calysta Yan, Joy E Arakaki, Matte Bailey, Ben Berry, Antoine Borensztejn, Eva M Brown, Sara Carlson, Julie A Cass, Basudev Chaudhuri, Kimberly R Cordes Metzler, et al. Integrated intracellular organization and its variations in human iPS cells. *Nature*, 613(7943):345–354, 2023. doi:10.1038/s41586-022-05563-7.
